# Role of Core Training in Judo Athletes: A Systematic Review

**DOI:** 10.3390/jcm15051897

**Published:** 2026-03-02

**Authors:** Nicola Marotta, Ennio Lopresti, Umile Giuseppe Longo, Andrea Demeco, Lorenzo Lippi, Francesco Zangari, Valerio Ammendolia, Michele Vecchio, Mario Vetrano, Marco Invernizzi, Alessandro de Sire, Antonio Ammendolia

**Affiliations:** 1 Physical and Rehabilitative Medicine, Department of Experimental and Clinical Medicine, University of Catanzaro “Magna Graecia”, 88100 Catanzaro, Italy; nicola.marotta@unicz.it; 2 Research Center on Musculoskeletal Health, MusculoSkeletalHealth@UMG, University of Catanzaro “Magna Graecia”, 88100 Catanzaro, Italy; andrea.demeco@unicz.it (A.D.); alessandro.desire@unicz.it (A.d.S.); ammendolia@unicz.it (A.A.); 3 Physical and Rehabilitative Medicine, Department of Medical and Surgical Sciences, University of Catanzaro “Magna Graecia”, 88100 Catanzaro, Italy; ennio.lopresti@studenti.unicz.it (E.L.); francesco.zangari@studenti.unicz.it (F.Z.); valerioammendolia95@gmail.com (V.A.); 4 Fondazione Policlinico Universitario Campus Bio-Medico, Via Alvaro del Portillo, 200, 00128 Rome, Italy; 5 Research Unit of Orthopaedic and Trauma Surgery, Department of Medicine and Surgery, Università Campus Bio-Medico di Roma, Via Alvaro del Portillo, 21, 00128 Rome, Italy; 6 Physical and Rehabilitative Medicine, Department of Health Sciences, University of Eastern Piedmont “A. Avogadro”, 28100 Novara, Italy; lorenzolippi.mt@gmail.com (L.L.); marco.invernizzi@med.uniupo.it (M.I.); 7 Dipartimento Attività Integrate Ricerca e Innovazione (DAIRI), Translational Medicine, Azienda Ospedaliera SS. Antonio e Biagio e Cesare Arrigo, 15121 Alessandria, Italy; 8 Department of Biomedical and Biotechnological Sciences, Section of Pharmacology, University of Catania, 95123 Catania, Italy; michele.vecchio@unict.it; 9 Physical and Medicine Rehabilitation Unit, “AOU Policlinico Rodolico”, 95123 Catania, Italy; 10 Physical Medicine and Rehabilitation Unit, Sant’Andrea Hospital, Sapienza University of Rome, 00189 Rome, Italy; mario.vetrano@uniroma1.it

**Keywords:** judoka, trunk, balance, spine, exercise, rehabilitation, martial arts

## Abstract

**Introduction**: Judo is a type of combat sport in which athletes must be able to constantly control their position and maintain a constant dynamic balance to respond to their opponent’s moves. In this scenario, the aim of this systematic review was to evaluate the role of core strength and stability in supporting balance, neuromuscular control, and functional performance-related determinants in judo athletes. **Methods**: PubMed, Scopus, and Web of Science databases were systematically used for articles published from inception to 4 April 2025, to identify any sort of manuscript indicating judo athletes as its population and core training approaches as the intervention (PROSPERO registry with the code: CRD420251032685). **Results**: Out of 401 studies, after the removal of 206 duplicates, we screened 195 records. Then, seven articles were included in the systematic review. We found that a strong core might improve balance and neurodynamic control. International-level judokas showed greater trunk extensor strength and less trunk angular displacement. Previous research suggests that core training improves physical fitness, balance, and lower limb recovery; moreover, the lack of core muscle strength might predispose athletes to injury, while solid core stability could ensure good support for the body to perform any movement in a balanced, coordinated, and functional manner. Core stability training and strengthening protocols might also decrease the risk of falling, which could have a beneficial effect on judoka athletes. **Conclusions**: Despite the wide variety of protocols used for core strengthening, it has been documented that a strong core might improve balance and neurodynamic control of movement during competition.

## 1. Introduction

Judo has been defined as a two-person confrontation combat sport where athletes are required to have control of their dynamic stance and motor gestures in order to counteract an opponent’s moves [1]. In particular, judo is a sport in which balance and imbalance and action and reaction alternate continuously, and where the objective of the two contenders is to obtain the greatest amount of points possible by throwing and/or knocking down the opponent, or to end a match by applying a grip or control technique [2].

Postural control is represented by the complex interaction between the sensory and motor nervous system, adapting balance to external stimuli to the spine [3]. The resulting postural orientation is given by the position of the trunk in response to gravity, and postural balance involves a coordinated sum of movement strategies in order to stabilize the body’s center of mass with respect to its base of support [4,5]. To maintain balance in judo competition, the postural system must try to maintain and manage the center of pressure (CoP) to avoid being pulled or thrown off balance by the opponent; thus, these continuous postural adaptations might occur through the integration of sensory receptors (somatosensory, visual, and vestibular), the musculoskeletal system, proprioceptive arrangement, and environmental conditions [6]. In judo, core muscles are fundamental for the transmission of force from the feet to the hands, a key principle for the effectiveness of both standing (tachi waza) and ground (ne waza) techniques, as they provide the stability and power necessary to throw or control an opponent, while also reducing the risk of back injuries [7]. Beyond its stabilizing role, the abdominal and trunk muscles also play a dynamic function in judo, actively contributing to trunk rotation, force generation, and energy transfer during throwing, gripping, and opponent manipulation [7,8,9]. From a biomechanical standpoint, the core acts as the kinetic chain’s central pivot, facilitating the seamless transmission of force between the lower and upper extremities. In the context of judo-specific maneuvers—such as gripping, pulling, and executing throwing techniques (nage-waza)—inadequate trunk stability may compromise force dissipation and exacerbate aberrant joint loading at distal segments, specifically the shoulder, knee, and ankle. Accordingly, deficits in core strength and neuromuscular control may elevate mechanical stress on these articulations, thereby increasing the susceptibility to both overuse pathologies and acute traumatic injuries [8,9,10,11,12,13,14,15].

Thus, one of the main goals of balance training is to prepare the body to have the most effective and efficient core possible, as the core has been identified as an anatomical structure composed of various muscles in the anterior (rectus abdominis), lateral (internal and external obliques), posterior (erector spinae, lumbar multifidus, and quadratus lumborum), upper (diaphragm), and lower (psoas iliacus) segments [8,9]. Henry Thomas [10] reported that the core could act as a link between the upper and lower body, supporting both symmetry and strength. Therefore, cross-linkage might explain how muscles are linked diagonally through the thoracolumbar fascia, allowing for efficient force transfer. Thomas [10] highlighted the importance of the lumbar–pelvic–hip complex as a central hub for movement, recommending exercises enhance hip stabilization and improve force transfer.

There is substantial evidence demonstrating a direct relationship between trunk muscle activity and lower limb kinematics [11]. Consequently, core stability is foundational to the efficient execution of nearly all motor activities, since a robust core provides critical functional support, enabling the body to perform movements with enhanced balance, coordination, and purposeful control [12]. Indeed, the importance of orthostatic balance and muscular strength has been highlighted in judokas; nevertheless, very few studies have been conducted on the impact of core stability in athlete performance [13,14].

A high degree of core stability is an essential prerequisite for injury prevention in judo. Despite the biomechanical advantages conferred by the core, the inherent risks associated with judo persist, necessitating a comprehensive approach to athlete safety [15]. The falls and projection techniques could expose athletes to specific injuries, such as those to the knee (particularly ACL tears) and the involvement of the shoulder and ankle [16]. This underscores the importance of targeted strength and conditioning and injury-prevention protocols, including core strengthening, to mitigate these risks and improve overall performance and safety in judo [17]; thus, in the sports medicine context, injury-prevention and neuromuscular conditioning strategies aim to reduce injury risk and enhance functional readiness prior to injury or competition [8,9,18,19,20,21].

Therefore, judo is, unfortunately, not an injury-free sport: the knee is the most involved, with 20% of judokas having experienced knee injury during the activity [18]. ACL injuries are the most severe injury type reported for judo athletes; in particular, this injury involves athletes who use the “Osotogari” and “Kosoto-gari” technique [22]. In addition to the knee, the shoulder and ankle are also often involved in injuries. The impact of a fall could cause, for example, a glenohumeral dislocation or a sprained ankle, even trauma resulting from arm locking techniques can lead to dislocations or fractures [23].

## 2. Materials and Methods

### 2.1. Data Sources and Searches

PubMed, Scopus, Web of Science databases were systematically searched for articles published from the inception until April 4th, 2025, according to each specific thesaurus, following this string strategy: “core” [All Fields] AND (“martial arts” [MeSH Terms] OR (“martial” [All Fields] AND “arts” [All Fields]) OR “martial arts” [All Fields] OR “judo” [All Fields]). This systematic review was conducted according to the guidance of Preferred Reporting Items for Systematic reviews and Meta-Analyses (PRISMA) [24]. The PRISMA checklist is included in the Supplementary Materials. No snowballing techniques or manual reference list searches were conducted.

The following systematic review was registered on the PROSPERO registry with the code: CRD420251032685.

### 2.2. Study Selection

After importing all search entries into a new Zotero (Center for History and New Media at George Mason University, Fairfax, VA, USA) library, the exclusion process began with two phases. First, duplicate records were automatically removed using Zotero’s built-in function; then, the remaining articles underwent a screening process to assess their eligibility for inclusion in this systematic review. This phase was conducted independently by two reviewers (EL and NM), who each created a separate list of included and excluded articles. These lists were then exported as a CSV file for analysis in Microsoft Excel. In cases where the two reviewers disagreed on an article’s eligibility, a third reviewer (AdS) was consulted to resolve the dispute and reach a final consensus, which enhanced the reliability of our selection process.

To guide our selection, eligibility criteria were established based on the PICOS (Participants, Intervention, Comparator, Outcome, Study design) model [25]. Thus, articles were considered eligible if they directly addressed the following parameters:

- (P) Participants: The papers had to exclusively involve judo athletes.
- (I) Intervention: We sought studies that investigated a core stability training and strengthening protocol, specifically core-specific interventions applied to judo athletes.
- (C) Comparator: Not required; when present, it was extracted and reported. The absence of control groups is considered a limitation.
- (O) Outcome measure: We considered any study reporting outcomes related to performance in judo athletes.
- (S) Study design: We considered experimental, quasi-experimental, and observational study designs (e.g., cross-sectional, longitudinal, and case–control studies). This inclusive approach allowed for a comprehensive understanding of the effects of core stability training, regardless of the level of control or the type of analysis.

To further refine our selection and maintain the focus and quality of the review, papers were systematically excluded based on the following criteria: Studies that included athletes from disciplines other than judo. Any administration of drugs or other physical agent modality treatments, i.e., interventions involving pharmacological medications or treatments like electrotherapy or ultrasound therapy were excluded to isolate the effects of core stability training. Unavailability of the full text (i.e., conference posters and abstracts); only studies with full-text availability were included to allow for a complete and detailed assessment of methodologies and results, preventing the reliance on potentially incomplete information. Most of the excluded studies investigated balance, strength, or injury epidemiology in judo athletes but did not include a core-specific training intervention, which was a mandatory inclusion criterion according to the PICOS framework. Other studies were excluded because they involved mixed martial arts populations, did not report performance-related outcomes, or were not available as full texts.

### 2.3. Data Extraction

Data from the selected studies were independently extracted by two reviewers (EL and NM) using a custom template. Any disagreements were resolved by consulting a third reviewer (AdS) to reach a consensus. Key information about the studies was summarized in tables (created with Microsoft Word 2021). The following data were extracted: (1) First author; (2) Publication year; (3) Nationality; (4) Age of study participants; (5) Population and the number of patients included; (6) Main limitations; (7) Main findings. Additionally, a narrative description provided a more detailed analysis of the included literature, especially for specific study details that could not be fully captured in the tables, which are thoroughly explained in Section 3.

### 2.4. Data Synthesis and Quality Assessment

The risk of bias assessment of the included studies was performed by two independent reviewers (EL and NM). The Joanna Briggs Institute (JBI) tool was used to assess the methodological quality of case–control studies, cohort studies, cross-sectional studies and case series. Each study was assessed according to predefined criteria [26]. The JBI model of evidence-based healthcare conceptualizes evidence-based practice as clinical decision-making that considers the best available evidence, the context in which care is delivered, client preference, and the professional judgment of the health professional. Any disagreements regarding its evaluation were resolved with the involvement of a third reviewer (AdS).

## 3. Results

### 3.1. Study Characteristics

**Figure 1:**
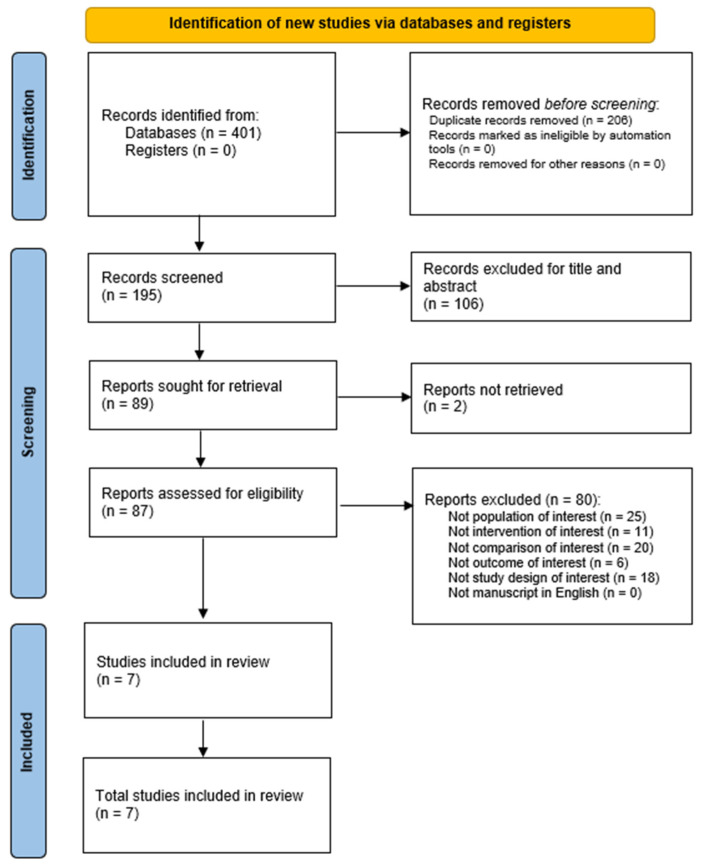
PRISMA flow chart.

The initial search returned 401 studies in the database searching reported by the authors. After the removal of 206 duplicates, we screened 195 records. After the screening for title and abstract and for full text, seven articles were included according to the eligibility criteria (for further details, see Figure 1).

### 3.2. Outcome Assessment

Seven articles from an initial search screening of 195 records were selected, as depicted in Table 1.

While the role of orthostatic balance and muscular strength has been examined in judo athletes, no attention has been paid to the effects of core strength on balance in judo athletes. An extreme heterogeneity in the methods of measuring core strength was immediately evident. For Yang et al. [30], surface electromyographic data, the centimeter difference between the dominant and non-dominant side, and the grip strength index were used. In Cai et al. [19], the ISOMED2000 isokinetic test system, the body composition test method, and the function inspection method were employed. Meanwhile, in Yasul et al. [20], the authors used three measurements for core strength (push-ups, planks, and sit-ups) at four time points (pre- and post-tests), six different single-leg hop tests (SLHTs) for lower extremity muscle strength, and the Y-balance test. In Barbado et al. [21], study assessments involved isokinetic tests and core stability measured using two protocols: sudden loading (to assess trunk responses to unexpected external perturbations) and stable and unstable sitting (to assess participants’ ability to control trunk balance). Finally, for Martins et al. [29], stabilometric analysis of center of pressure behavior parameters and baropodometric analysis (peak pressure obtained during a 30 s acquisition period and expressed based on the area of the foot) were carried out.

In summary, the outcomes measured were varied and not always directly relevant to on-mat judo performance. While some studies measured specific performance indicators like (YBT) and lower limb strength (SLHT), others focused on more general metrics like baropodometric analysis, isokinetic strength, and even EMG data.

An extreme variety of methods was also accompanied by a heterogeneity of the populations examined across the various studies. For instance, Cai et al. [19] examined a single athlete (a Chinese judo team member with a lower limb injury). Yasul et al. [20], on the other hand, evaluated two groups of judo athletes, divided by gender. Yang et al. [30] analyzed two groups: one consisting of nine male judo athletes from competitive sports schools, and the other comprising nine ordinary college students without prior sports training experience. Only Martins et al. [29] established two homogeneous groups, each with nine athletes from the University of Southern Santa Catarina (UNISUL). Lastly, Barbado et al. [21] considered 11 international-level and 14 national-level judo practitioners. However, a significant limitation of their study was that it did not assess the relationship between trunk parameters and specific judo skills, focusing instead on overall sport performance criteria (i.e., ranking, level of competition, etc.).

### 3.3. The Role of Core Training in Judo

In all the examined studies, core strengthening programs were implemented, though the protocols varied. Martins et al. [29] analyzed two groups (experimental, *n* = 9; control, *n* = 9) that followed the same core strengthening protocol (30 min sessions, twice weekly for five consecutive weeks), with evaluations conducted in both eyes-open and eyes-closed conditions. Baropodometric analysis revealed a statistically significant difference in the right/left foot relative pressure ratio between groups post-intervention in the eyes-open evaluation (*p* < 0.05). For stabilometric parameters (latero-lateral and anteroposterior CoP oscillations), the eyes-open evaluation showed a statistically significant increase in latero-lateral oscillation in the experimental group (width, *p* < 0.05) but not in the control group (*p* > 0.05). Barbado et al. [21] assessed the role of core training in 14 national-level and 11 international-level judokas. They found that international-level judokas exhibited significantly higher trunk extensor isokinetic strength (*p* < 0.05) and less trunk angular displacement after anterior trunk loading (*p* < 0.05) compared to national-level judokas. Yang et al. [30] investigated the impact of core strengthening on physical fitness by dividing 18 male athletes into two groups: nine judo athletes from competitive sports schools and nine ordinary college students without prior sports training experience. A core strengthening protocol was integrated to analyze the relationship between judo athletes’ physical conditioning and the structural characteristics of their technical training. Results indicated that core training could improve the physical fitness of young judo athletes. Significant differences were noted in the side grip strength index between the dominant side of the judo group and the non-judo group (*p* < 0.05), as well as in the forearm circumference of the dominant side, the maximum grip value of the dominant side, and the EMG data between the two groups. Yasul et al. [20] also demonstrated the beneficial effects of core training using a two-group pre-test/post-test experimental design with repeated measures. This study involved 12 female judo athletes (aged 12–18 years) and 10 male judo athletes (aged 12–17 years). After three core strength measurements (push-ups, planks, and sit-ups) at four time points (pre- and post-tests), six different single-leg hop tests (SLHTs) for lower extremity muscle strength, and the Y-Balance Test (YBT) for balance, the results showed comprehensive improvements. Both right and left leg YBT scores considerably rose in post-test assessments, limb symmetry index (LSI) scores increased, and push-up, plank, and sit-up scores improved. For the dominant (D) leg hop for distance, male and female judokas’ scores (SLD = single-leg distance; THD = triple hop distance; MSTHD = medial side triple hop distance; MRHD = medial rotation hop distance; CHD = crossover hop distance) generally increased (with the exception of female CHD scores). Both male and female athletes also demonstrated better performance in the single-leg 6-m hop (SL6MD).

Lastly, it should also be reported that the case study performed by Cai et al. [19], involving a 21-year-old male judo athlete (183 cm, 95.7 kg) who experienced a lower limb injury (diagnosed in October 2019 after intense training), evaluated a three-stage core training program. The athlete underwent a supervised core-stability-focused rehabilitation program, which was the component analyzed in this report, and showed good recovery in lower limb function (*p* < 0.05) and physical fitness level (*p* < 0.05). Although both static (e.g., planks, isometric holds) and dynamic (e.g., trunk rotations, unstable-surface exercises) core exercises were used, no study directly compared these modalities. Nevertheless, no evidence-based recommendation can be made regarding the superiority of static or dynamic core training for judo-specific performance.

### 3.4. Risk of Bias

In the present systematic review, we used the JBI Critical Appraisal Checklist for quasi-experimental studies to assess the risk of bias of the included studies, as depicted in Table 2.

The table highlights that the management of confounding factors was a common weakness across many included studies [20,21,27,28,30]. This is a critical aspect for internal validity, as uncontrolled factors can influence results and lead to incorrect conclusions about the exposure–outcome relationship. Some studies [20,21] also show issues with initial group similarity or the “free of outcome at start” condition, which are fundamental for establishing causality. Lastly, Martins et al. [29] appear to have the most robust methodology among those assessable with this checklist, though it still has room for improvement in managing confounding variables.

## 4. Discussion

The aim of our study was to evaluate whether and how core training might influence performance in judo. Although the term “core” is typically used to identify the muscles essential for maintaining functional stability of the trunk and hip joints against factors that alter their balance, and despite evidence that static elements (bones and soft tissues) contribute to core stabilization, its maintenance is largely attributed to the dynamic function of muscular elements. It has also been documented that a weak core leads to ineffective movements and predisposes individuals to injury, whereas a strong core provides a stable base for the body to perform any movement in a balanced, coordinated, and functional way [31,32].

Despite this consensus on the key role of core, an extreme variety of materials, methods, and results were highlighted across the studies reviewed [33]. Martins et al. [29] examined the most homogeneous population, dividing eighteen athletes from the UNISUL into two groups. In contrast, Yasul et al. [20] divided athletes into two groups of 18 judokas: nine high-level judo athletes and nine university students without prior sports experience. Aboelwafa et al. [28] included 24 Egyptian judokas in the study, to form two groups: an experimental group (12 players), who were trained via core-muscle-specific intervention, and a control group of 12 players. Cai et al. [19] evaluated only a single case report involving a male athlete from the Chinese judo team. Barbado et al. [21], assessed differences in trunk muscle function between eleven international and fourteen national judo athletes. Lastly, Yasul et al. [20] included twelve female judo athletes aged 12–18 in one group and ten male judo athletes aged 12–17 in another, separated by gender; as well as Akinoglu et al. [27], that assessed twenty judo athletes (11 males, 9 females) aged 13–19 years, who participated in the study.

Core training in judo appears to positively influence several aspects of performance and stability [27,34]. Baropodometric analysis revealed a significant alteration in the right/left foot pressure ratio, indicating changes in load distribution, while increased CoP oscillation is traditionally associated with postural instability. In the specific context of athletic conditioning, it might instead reflect an enhanced adaptive capacity of the neuromuscular system. Therefore, this increased lateral variability might suggest a more flexible postural strategy, wherein the athlete effectively utilizes a wider area of the base of support to maintain dynamic balance; this is even more evident in high-demand sports like judo, where ‘functional variability’ becomes essential due to the requirement for constant adaptation to external perturbations [29,35].

Although no differences were found in trunk flexor isokinetic strength, international-level judokas demonstrated higher trunk extensor strength and less trunk angular displacement under anterior loading, crucial elements for stability during grappling and throws [21]. Notably, there was a significant recovery in lower limbs and fitness levels, accompanied by improved performance in balance tests, core strength (push-up, plank, and sit-up), and lower limb power [20]. These findings, along with significant differences in dominant-side grip strength and forearm circumference between judo and non-judo groups, highlight how specific core training contributes not only to trunk stability and strength but also to functional improvements essential for the unique demands of judo; this can be explained by improved force transmission along the kinetic chain, whereby enhanced trunk stability allows for a greater expression of distal force at the forearm and hand during gripping.

In this context, the core training programs themselves also varied significantly. For Yang et al. [30], an unspecified core muscle strengthening program was included in the experimental control group, similar to the case study in Cai et al. [19]. Martins et al. [29] proposed a core strengthening protocol for the experimental group, which involved 30 min sessions twice a week for five consecutive weeks. Barbado et al. [21], without specific core training, demonstrated that international-level judokas exhibited greater trunk extension strength and better responses after a sudden anterior trunk load compared to national-level judokas, suggesting that core strengthening is essential for elite judo athletes. Only Yasul et al. [20] clearly illustrated their core reinforcement schedule with a protocol divided by days and weeks.

Only two [29,30] of the five studies provided a control group, which makes it difficult to isolate the true effect of the core training intervention from other factors. Moreover, Yang et al. [30] and Yasui et al. [20] administered non-supervised protocols, which might have introduced a potential for inconsistency in training execution and adherence, further compromising the reliability of the results.

While some studies measured specific performance indicators like balance (YBT) and lower limb strength (SLHT), others focused on more general metrics like baropodometric analysis, isokinetic strength, and even EMG data.

In this context, injury-prevention and neuromuscular conditioning strategies—including remotely supervised core training programs—may represent a valuable tool to maintain neuromuscular conditioning, especially in periods of reduced training availability, such as the post-COVID-19 scenario [6,36,37,38,39,40,41,42].

Lastly, it is also very important to consider that skeletal maturation plays a crucial role in the development of postural imbalances in young judokas, as the asynchronous growth of bones and soft tissues during puberty can transiently alter joint alignment, neuromuscular control and load distribution, thereby increasing susceptibility to compensatory postures and asymmetries typical of unilateral throwing techniques [43,44].

In this context, a recent RCT evaluated the efficacy of a trainer-supervised judo-specific injury prevention warm-up program on overall injury prevalence, showing that the Injury Prevention and Performance Optimization Netherlands (IPPON) intervention did not significantly reduce the overall and severe injury prevalence; however, von Gerhardt et al. suggested that the IPPON intervention be considered as an useful alternative to regular judo warm-up, given the high adherence and the positive clinical experiences of trainers and athletes [44]. At the same time, it has been showed that both unstable and stable resistance training effectively improved maximal strength and performance in athletes, avoiding ligament or muscle injuries and trauma particularly in COVID-19 era [40,45,46].

Therefore, the biomechanical functionality of the core represents a cornerstone of judo performance, transitioning from a mere anatomical construct to a dynamic muscular system essential for maintaining stability during complex maneuvers such as *nage-waza* [29]. Clinical evidence suggests that a robust core facilitates optimized load distribution and superior trunk extensor strength—key discriminators between elite and national-level athletes—while simultaneously mitigating injury risks associated with inefficient movement patterns [43]. Furthermore, specific interventions have demonstrated tangible improvements in functional parameters, including baropodometric balance and grip strength, which are vital for managing the asymmetrical loads typical of unilateral throwing techniques [27,29,43]. Despite the observed clinical benefits, the literature exhibits significant heterogeneity in training protocols and supervision, highlighting the need for standardized “gold standard” programs. Emerging strategies, such as the IPPON warm-up and telerehabilitation, further underscore the importance of a multifaceted approach to maintaining both physical resilience and postural symmetry during critical developmental stages [44].

Nevertheless, our review has several limitations. Firstly, the limited number of included studies (*n* = 7) and the small sample sizes, with a maximum of only 25 participants in the largest cohort [21]. Secondly, a significant source of concern is that only two out of the seven studies included a control group, which limits the ability to definitively attribute observed improvements solely to the core training intervention. Thirdly, the systematic review revealed high heterogeneity in the design and implementation of training protocols, including variations in duration, intensity, and the presence of unsupervised sessions, which may introduce significant bias. In this scenario, the risk of bias assessment indicated substantial methodological weaknesses across the included studies, further weakening the strength of the evidence. Moreover, the frequent absence of control groups across the literature is acknowledged as a significant limitation of the current evidence base. Fourthly, there was a limited comparison between core stability improvements and actual on-field sports performance, as most outcomes focused on laboratory-based biomechanical measures. Lastly, the lack of a meta-analysis, combined with the absence of snowballing techniques or manual reference searches, may have further limited the breadth of the search strategy and major findings.

## 5. Conclusions

The main finding of this systematic review is that core strength and stability may be associated with improved balance, trunk control and functional performance-related outcomes in judo athletes. However, the definition, assessment and training of the ‘core’ varied widely across studies, preventing direct comparison and the identification of an optimal training model. Future studies should focus on larger, more standardized sample sizes, include appropriate control groups, and use judo-specific performance metrics to better understand the true impact of core training on the sport. The observed heterogeneity highlights the need for a consensus on optimal training protocols and measurement tools to advance this area of research.

## Figures and Tables

**Table 1:** Main characteristics of the studies included in the present systematic review.

Article	Nation	Study Group	Intervention	Outcomes	Limitations	Main Findings
Akinoglu et al., 2017 [27]	Turkey	Twenty judo athletes (11 males, 9 females) aged 13–19 years participated in the study.	All athletes were requested to perform an 8-week core endurance exercise program with one set of 10 repetitions, two times a day, with static trunk flexion endurance, static trunk extension endurance, and static side bridge tests.	Hip flexion and extension muscle strength were measured isokinetically. Trunk muscle endurance was assessed before and after training.	Study failed to account for the athletes’ experience level, relying on a single outcome measure. Moreover, intervention lacked a control group for comparison and placed an overriding focus on gender differences.	The study’s findings indicated that males exhibited superior hip flexor and extensor muscle strength. Post-training analysis revealed that the hip flexion/extension ratio in both genders had been brought into the normal range (59–74%). Regarding trunk endurance, all participants demonstrated an improvement across all trunk muscle endurance test parameters following the training intervention. Notably, male judo athletes consistently achieved significantly higher scores on all trunk muscle endurance tests, with the exception of the extensor endurance test.
Aboelwafa et al., 2022 [28]	Saudi Arabia	24 Egyptian judo players participated in the study to form two groups: an experimental group (12 players), who trained core muscles, and a control group (12 players), who trained various muscle groups.	Eight core exercises, 3 sets of 15 repetitions each: knee bend, alternate leg, bridge, plank, oblique plank, single and double crunches, and toe touch with knees bent.	The balance test (Flamingo) from the European test battery was used to measure the static balance of the players. In this test, each player stands on one foot on a crossbar (1 inch wide, 1 inch high and 20 inches long) and holds the other foot by the ankle using the hand.	The level of experience of the athletes is not recognized. Only one study outcome is used. This suggests the research might be too narrow, focusing only on a single measure without considering other potential effects or variables.	In the study, the experimental group showed significantly greater progress in the Flamingo test than the control group. This difference is attributed to the effectiveness of core strengthening exercises in improving static balance.
Barbado et al., 2016 [21]	Spain	International (*n* = 11) and national (*n* = 14) level judo practitioners (judokas), black belt with more than 7 years of experience and a workout frequency of 3–5 days per week.	Supervised two distinct approaches: the first involved an isokinetic test with pneumatic traction for load application, the second utilized a seated trunk training protocol with real-time biofeedback of the participants’ center of pressure.	Trunk strength and endurance seated in the dual position back extension/flexion attachment of an isokinetic dynamometer with trunk stability parameters (sudden loading, stable and unstable sitting).	Lack of assessment for physical performance and their impact on judo performance. Failure to evaluate the relationship between trunk parameters and specific judo performance indicators.	No differences were found in trunk flexor isokinetic strength between groups. However, international-level judokas exhibited significantly higher trunk extensor isokinetic strength and less trunk angular displacement after anterior loading.
Cai, 2022 [19]	China, Brazil	A 21-year-old male of the Chinese National Judo Team (183 cm tall and weighing 95.7 kg), diagnosed with an injury to the collateral ligament of his left ankle.	A supervised program of 3 sets of planks, side plank, opposite arm and leg, all fours, double side jackknife, gluteal bridge (2 times a week for 8 weeks)	Isokinetic muscle strength test method (ISOMED2000 isokinetic test system), body composition test method (Korean InBody3.0 body composition analyzer).	Case report	A recovery in fitness level was noted. Lower limbs were more coordinated, and muscular symmetry difference was less than 15%. Moreover, the authors reported that muscles demonstrated a low ratio of abdominal to back muscles.
Martins et al., 2019 [29]	Brazil, France	Athletes (*n* = 18) from a Brazilian university. Green or higher rank belt, with at least three years of continuous training twice a week.	Only experimental group (*n* = 9) performed supervised 10-day program: 3 sets of abdominal butterfly, abdom. obliques, climbing, trunk extensions, and front plank	Stabilometric and baropodometric analysis performed at baseline and after 5 weeks of core strengthening; the athletes were evaluated in two situations: eyes-open and eyes-closed.	Small sample size; fatigue may have influenced the results.	Baropodometric analysis presented a significant difference in latero-lateral foot pressure comparison between groups post-test with eyes open (*p* < 0.05). Stabilometric data reported a significant rise in latero-lateral oscillation in the experimental group during eyes-open evaluation, while a non-significant variation in the control group.
Yang, 2022 [30]	China, Brazil	Two groups: male judo athletes (*n* = 9) and a control group of college students (*n* = 9) without sports training experience.	Non-supervised program of 3 sets of abdominal crunch, vertical leg crunch, jack knife supine knee side-to-side, reverse crunch plank jack (2 times a week for 8 weeks).	Surface EMG grip strength index [(c/T ratio of myoelectricity to muscle strength (v/kg); center frequency CF (Hz)], body shape of subjects.	(1) Small sample size. (2) No analysis of possible confounding factors.	The authors reported a significant difference in side grip strength in the dominant-side comparison; moreover, a significant difference in the forearm circumference of the dominant side. A significant difference in the maximum grip value of the dominant side and significant differences in EMG data between groups were demonstrated.
Yasul et al., 2023 [20]	Türkiye	Two-group pre-test/post-test experimental design: female judo athletes (*n* = 12) aged 12–18 years and male judo athletes (*n* = 10) aged 12–17 years.	Non-supervised 6 weeks of core exercise (3 sessions per week): plank, push-up, reverse crunch, bird dog, basic squat, toe taps, squat leg raise.	Three measurements repeated at multiple time points within the pre- and post-intervention phases, six different single-leg hop tests (SLHTs) for lower extremity muscle strength and YBT (Y Balance Test) for balance.	(1)No control group except the core strength group. (2) Males and females were evaluated separately. (3) Small sample size. (4) Diet and meals were not monitored.	Both right and left YBT scores and limb symmetry index (LSI) scores increased. Push-up and plank times, along with sit-up scores, also improved. For the dominant leg hop for distance, most male and female judokas’ scores increased, with similar improvements seen in the non-dominant leg hop. Male and female judokas also demonstrated better performance in the 6 m Timed Hop Test.

**Table 2:** Joanna Briggs Institute risk of bias quality assessment.

Study	Q1	Q2	Q3	Q4	Q5	Q6	Q7	Q8	Q9	Q10	Q11
Akinoglu et al. [27]	NA	Partially	Partially	No	No	Yes	Partially	No	NA	No	Yes
Aboelwafa et al. [28]	Yes	Yes	Partially	No	No	Yes	Partially	No	NA	No	Yes
Barbado et al. [21]	Yes	Yes	Yes	Partially	Partially	No	Yes	No	No	NA	Yes
Cai [19]	NA	NA	NA	NA	NA	NA	NA	NA	NA	NA	NA
Martins et al. [29]	Yes	Yes	Yes	Partially	No	Yes	Yes	Yes	Yes	NA	Yes
Yang et al. [30]	Yes	Yes	Yes	No	No	Yes	Yes	Yes	Yes	NA	Yes
Yasul et al. [20]	Yes	No	Yes	No	No	Yes	Yes	Yes	Yes	NA	Yes

## Data Availability

The data supporting the findings of this systematic review are contained within the article. The datasets extracted and analyzed during the current study are available from the corresponding author upon reasonable request.

## Supplementary Materials

The following supporting information can be downloaded at: https://www.mdpi.com/article/10.3390/jcm15051897/s1, PRISMA checklist is available in the Supplementary Materials section.

## Abbreviations

CoP	Center of pressure
PICOS	Participants, Intervention, Comparator, Outcome, Study design
JBI	Joanna Briggs Institute
SLHT	Single-leg hop test
SLHT	Lower limb strength
UNISUL	University of Southern Santa Catarina
YBT	Y-balance test
LSI	Limb Symmetry Index
SL6MD	Single-leg 6-m hop
IPPON	Injury Prevention and Performance Optimization Netherlands
